# Altered spatiotemporal brain dynamics of interoception in behavioural-variant frontotemporal dementia

**DOI:** 10.1016/j.ebiom.2025.105614

**Published:** 2025-02-22

**Authors:** Jessica L. Hazelton, Gabriel Della Bella, Pablo Barttfeld, Martin Dottori, Raul Gonzalez-Gomez, Joaquín Migeot, Sebastian Moguilner, Agustina Legaz, Hernan Hernandez, Pavel Prado, Jhosmary Cuadros, Marcelo Maito, Matias Fraile-Vazquez, María Luz González Gadea, Yasir Çatal, Bruce Miller, Olivier Piguet, Georg Northoff, Agustin Ibáñez

**Affiliations:** a Latin American Brain Health Institute (BrainLat), Universidad Adolfo Ibáñez, Santiago, Chile; b Cognitive Neuroscience Centre (CNC), Universidad de San Andres, Buenos Aires, Argentina; c The University of Sydney, Brain and Mind Centre, School of Psychology, Sydney, Australia; d Cognitive Science Group, Instituto de Investigaciones Psicológicas (IIPsi, CONICET-UNC), Facultad de Psicología, Universidad Nacional de Córdoba, Córdoba, Argentina; e Facultad de Matemática Astronomía y Física (FaMAF), Universidad Nacional de Córdoba, Córdoba, Argentina; f Escuela de Fonoaudiología, Facultad de Odontología y Ciencias de la Rehabilitación, Universidad San Sebastián, Santiago de Chile, Chile; g Advanced Centre for Electrical and Electronic Engineering (AC3E), Universidad Técnica Federico Santa María, Valparaíso, Chile; h Grupo de Bioingeniería, Decanato de Investigación, Universidad Nacional Experimental del Táchira, San Cristóbal, 5001, Venezuela; i Life Span Institute, University of Kansas, Lawrence, KS, USA; j National Scientific and Technical Research Council (CONICET), Buenos Aires, Argentina; k Mind, Brain Imaging and Neuroethics, Institute of Mental Health Research, University of Ottawa, Ottawa, Canada; l Global Brain Health Institute (GBHI), University of California San Francisco (UCSF), California, USA; m Global Brain Health Institute (GBHI), Trinity College Dublin, Dublin, Ireland; n Mental Health Centre, Zhejiang University School of Medicine, Hangzhou, Zhejiang, People's Republic of China; o Centre for Cognition and Brain Disorders, The Affiliated Hospital of Hangzhou Normal University, Hangzhou, People's Republic of China

**Keywords:** Interoception, ACW, bvFTD, Dementia, Brain dynamics, Allostasis

## Abstract

**Background:**

Dysfunctional allostatic-interoception, altered processing of bodily signals in response to environmental demands, occurs in behavioural-variant frontotemporal dementia (bvFTD) patients. Previous research has not investigated the dynamic nature of interoception using methods like intrinsic neural timescales. We hypothesised that longer intrinsic neural timescales of interoception would occur in bvFTD patients, evidencing dysfunctional allostatic-interoception.

**Methods:**

One-hundred and twelve participants (31 bvFTD patients, 35 Alzheimer's disease patients, AD and 46 healthy controls) completed a well-validated task measuring cardiac-interoception and exteroception. Simultaneous EEG and ECG were recorded. Intrinsic neural timescales were measured via the autocorrelation window (ACW) of broadband EEG signals from each heartbeat and a time-lagged version of itself. Spatiotemporal clustering analyses identified clusters with significant between-group differences in each condition. Voxel-based morphometry was used to target the allostatic-interoceptive network. Neuropsychological tests of cognition and social cognition were assessed.

**Findings:**

In bvFTD patients, longer interoceptive-ACWs than controls were observed in the bilateral fronto-temporal and parietal regions. In AD patients, longer interoceptive-ACWs than controls were observed in central and occipitoparietal brain regions. No differences were observed during exteroception. In bvFTD patients only, longer interoceptive-ACW was linked to worse sociocognitive performance. Structural neural correlates of interoceptive-ACW in bvFTD involved the anterior cingulate, insula, orbitofrontal cortex, hippocampus, and angular gyrus.

**Interpretation:**

Our findings suggest a core allostatic-interoceptive deficit occurs in people with bvFTD. Further, altered interoceptive intrinsic neural timescales may provide a neurobiological mechanism underpinning the complex behaviours observed in bvFTD patients. Our findings support synergistic models of brain disease and can inform clinical practice.

**Funding:**

All funding sources are reported in the Acknowledgements.

**Research in context**

**Evidence before this study**

Emerging evidence suggests that altered allostatic-interoceptive processing occurs in neurodegeneration, with particular relevance for behavioural variant frontotemporal dementia. Previous research, however, has used static measures of interoceptive processes, such as neuroanatomical correlates of behavioural tasks. These methods do not consider the inherently dynamic nature of allostatic-interoception. Further, it is currently unknown how these processes may be impacted in neurodegeneration and the relationship with behavioural patterns observed in the disease.

**Added value of this study**

In a well-characterised cohort of behavioural variant frontotemporal dementia and Alzheimer's Disease patients, we employed a novel measure of brain activity that captures the dynamic processing on interoceptive information. Our results suggest that altered intrinsic neural timescales of interoception occur in behavioural variant frontotemporal dementia and Alzheimer's disease, unrelated to previously used static measures of interoception. In behavioural variant frontotemporal dementia only, these disrupted interoceptive intrinsic neural timescales were associated with sociocognitive performance and neural correlates within the allostatic-interoceptive brain network.

**Implications of all the available evidence**

Taken together, our results support a core allostatic-interoceptive deficit in behavioural-variant frontotemporal dementia. This core deficit could reflect a neurobiological mechanism that may underlie the outward behavioural changes observable in this disease. Our work supports synergistic models of brain health and disease, has implications for earlier diagnosis and disease monitoring, and may inform potential interventions.

## Introduction

Interoception impairment (i.e., difficulties in the sensing, perceiving, and anticipating of signals arising from within the body) is a core feature that can occur in multiple clinical conditions.^1^, ^2^, ^3^ Interoceptive deficits are observed in brain diseases involving impairments in autonomic regulation and visceral (e.g., cardiac) signalling.^4^^,^^5^ Most research to date, however, has been based on neuroanatomical correlates with fMRI or studying the heart-evoked potential (HEP).^6^, ^7^, ^8^ Despite their merit, these approaches are blind to the spatiotemporal dynamics of interoception operating across timescales,^9^^,^^10^ or how these dynamics are associated with neurocognitive profiles. Recently, a unified framework has been proposed to overcome these challenges, combining the predictive coding theory of allostatic interoceptive overload (PAIO) and the intrinsic neural timescales (INT) theory.^11^ The PAIO-INT framework provides a synergistic model to understand brain health and disease,^12^ however, to date, limited empirical evidence directly testing this framework exists.

The PAIO considers the coding of internal (interoceptive) and external (exteroceptive) signalling, grounded in allostasis and interoception.^11^ Allostasis (i.e., the brain's continual prediction, anticipation, and adaptation to meet the body's needs before they arise or become urgent)^13^ is deeply intertwined with interoception.^3^ Allostatic-interoception is underpinned by the Allostatic-Interoceptive Network (AIN), a large-scale domain-general network including the anterior cingulate cortex, insula, orbitofrontal cortex, amygdala, thalamus, hippocampus, ventral striatum, and angular gyrus.^1^^,^^3^^,^^14^ The INT theory proposes that different brain regions operate on a hierarchy of intrinsic timescales, which are used to process and actively shape the multitude of inputs the brain receives from the ever-changing internal and external environment.^11^ One way to directly measure intrinsic neural timescales is via autocorrelation windows (ACW).^15^ The ACW is the correlation between a signal and a time-lagged version of itself and represents the stability of a signal.^15^ Within the combined PAIO-INT framework, shorter ACW of interoceptive signals would enable rapid processing of interoceptive information, whereas longer ACW of interoceptive signals would represent delayed processes, potentially associated with AIN dysfunctions.^11^

Behavioural-variant frontotemporal dementia (bvFTD) provides an opportunity to investigate the PAIO-INT framework via a lesion model approach.^11^^,^^14^^,^^16^ Accumulating evidence suggests that bvFTD is associated with core interoceptive deficits, allostatic overload and autonomic dysfunction^17^, ^18^, ^19^, ^20^, ^21^, ^22^, ^23^ linked to sociocognitive impairments.^17^^,^^19^ Moreover, both atrophy and functional connectivity alterations observed in people with bvFTD^14^^,^^17^^,^^24^ substantially overlap with the AIN.^3^ Some evidence suggests that Alzheimer's disease (AD) also disrupts interoceptive and allostatic processes,^18^ as well as cardiovascular indices.^23^^,^^25^ The nature and extent of these disruptions, however, is reduced compared to previous observations in people with bvFTD. The transient and dynamic nature of these interoceptive deficits in people with bvFTD or AD is unclear, and previous evidence of interoceptive deficits has been largely derived from traditional, static, or behavioural measures.^17^, ^18^, ^19^

Interoception, however, is inherently dynamic, and involves transient spatiotemporal processes.^11^ Neurodegenerative processes may disrupt the brain's intrinsic neural timescales in different regions, providing a mechanism underlying the heterogenous symptoms often observed. For instance, people with bvFTD display socially inappropriate behaviour, emotional blunting, and apathy (all likely related to interoception deficits)^11^ are commonly observed symptoms.^26^, ^27^, ^28^ This disrupted intrinsic neural processing within the AIN may imbalance interoceptive and exteroceptive signals,^11^ potentially explaining the maladaptive responses to environmental demands observed in people with bvFTD.^14^ In people with AD, the disruption of the brain's intrinsic temporal irreversibility (i.e., the temporal asymmetry of brain dynamics)^29^ has been associated with cognitive decline, although this study was not specific to interoception. Further, emerging evidence suggests that brain and heartbeat dynamics can shift as a function of healthy ageing and in other neurodegenerative conditions such as Parkinson's disease.^30^^,^^31^ Beyond these emerging studies, the impact of altered spatiotemporal brain dynamics on interoception in people with bvFTD and AD remains poorly understood. To our knowledge, no prior research has investigated intrinsic neural timescales of interoception compared to exteroception in patients with bvFTD or AD. Additionally, it appears that there has been no investigation linking altered timescales to behavioural or sociocognitive impairments in these conditions. Moreover, we believe that no study has combined the spatiotemporal dynamics of interoception with the anatomical correlates of the AIN. Finally, to our knowledge, the ACW has not been applied to understanding the different spatiotemporal dynamics of interoception and exteroception in brain health or disease.

**Fig. 1:**
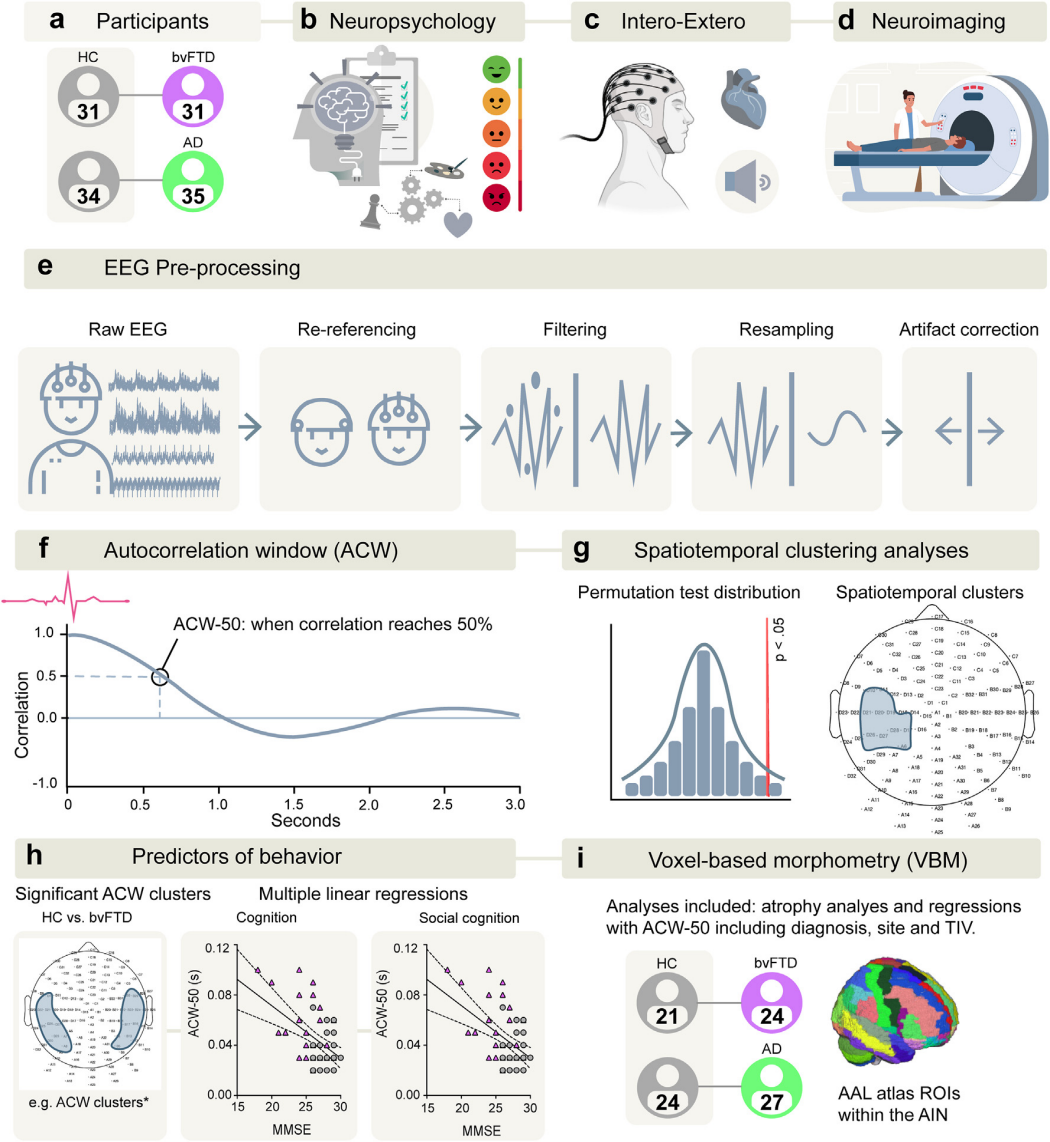
**Experimental workflow diagram**. a) Participants were matched on demographic variables to create bvFTD-HC and AD-HC tandems. b) Neuropsychological tests were conducted including cognition, executive functioning, and social cognition measures. c) Participants completed a validated interoception task with simultaneous EEG recording. d) 3D T1 scans were attained for a subsample of participants. e) EEG preprocessing steps included re-referencing channels, filtering, resampling and artifact correction per standardised protocols. f) ACWs were calculated starting on each heartbeat over a 3-s window. Plotted is an example of the Autocorrelation function and the time points where ACW of the heartbeat reaches 50%. g) Spatiotemporal clustering analyses were conducted. Permutation-based testing (5000 permutations) was used to detect significant clusters. h) Multiple linear regressions were conducted to predict sociocognitive functioning using the significant ACW clusters in each group, together with demographic variables and diagnostic status. i) Voxel-based morphometry analyses were conducted to identify atrophy patterns between groups. Then, regression models were conducted between the ACW and the AIN. Masks were created based on the AAL3 for the AIN-core: anterior cingulate cortex, insula, amygdala; and AIN-extended: AIN-core plus Mid cingulate, orbitofrontal cortex, thalamus, hippocampus, angular gyrus, and ventral striatum. Covariates included diagnosis, site, and TIV. Sample sizes are reported in a) and in i). Abbreviations: AAL, Automated Anatomical Labelling; ACW, Autocorrelation window; AD, Alzheimer's disease; bvFTD, behavioural variant frontotemporal dementia; HC, Healthy Controls; ROI, Regions of Interest; TIV, Total intracranial volume. Some elements were created with Biorender.com.

This work aims to bridge these gaps. First, we investigated how INT scales in interoception and exteroception differ in people with bvFTD and AD. All participants performed a well-validated heartbeat tracking task,^18^, ^19^, ^20^^,^^32^, ^33^, ^34^ where attention was directed to either interoceptive (i.e., heartbeat) or exteroceptive (i.e., recorded heartbeat) cues. Simultaneous high-density 128-channel EEG was recorded. Using a data-driven approach, we aimed to uncover spatiotemporal patterns across the whole brain associated with differences in ACW between patients and controls during interoception or exteroception. To achieve this aim, we calculated the ACW associated with each R-wave, which represents a measure of EEG signal decay over time (Fig. 1). Unlike the HEP, which typically occurs 250–550 ms post R-wave,^6^ we anticipated that the ACW associated with each heartbeat would occur on a much faster timescale to reach 50% (or 0.5 correlation value) well before 250 ms, in line with previous studies investigating ACW of EEG signals.^35^ We hypothesised that longer ACW would be observed in patient groups than controls due to disrupted intrinsic neural timescales during interoception. We also explored the relationship between ACW and metrics related to interoception (e.g., interoceptive accuracy, heart rate variability, and HEP). Second, we aimed to explore the relationship between these altered INT and sociocognitive functioning in each disease. We anticipated that the altered INTs in patients with bvFTD would be associated with sociocognitive dysfunction, supporting an allostatic interoceptive overload interpretation.^17^ Finally, we linked these altered temporal brain dynamics to neuroanatomical changes within the AIN, to provide insights into disease mechanisms. We expected that altered INT would be associated with brain regions within the AIN,^3^^,^^14^ such as the insula, anterior cingulate cortex, orbitofrontal cortex, amygdala, thalamus, hippocampus, ventral striatum, and angular gyrus.

## Methods

### Participants

Participants (n = 112) included 31 patients with bvFTD, 35 patients with AD, and 46 healthy controls (HC). Data were obtained from the BrainLat database.^36^ Patients were diagnosed by expert clinicians in line with current diagnostic criteria for probable bvFTD or AD.^37^^,^^38^ Recruitment and diagnoses were conducted in clinical centres by a multidisciplinary team as part of an ongoing multi-centric protocol. Multidisciplinary examinations supported diagnoses in line with the Multi-Partner Consortium to Expand Dementia Research in Latin America standardised protocol.^39^^,^^40^ Exclusion criteria included the history of other neurologic disorders, psychiatric conditions, primary language deficits, or substance abuse. HC were demographically matched to each patient sample using *R* MatchIt^41^ to create two tandem groups for comparison (HC-bvFTD n = 31; HC-AD n = 34). Patterns of differences in grey matter integrity in bvFTD vs controls, and AD vs controls were consistent with atrophy patterns previously reported in each disease (Supplementary Tables S1 and S2).

### Ethics

All participants or their caregivers provided informed written consent in line with the Declaration of Helsinki. The study was approved by the Ethics Committees of the involved institutions (Argentina: INECO-Centro de Psicología Médica San Martín de Tours: FWA00028264; Chile: Geroscience FONDAP. Universidad de Chile FWA00029236 and Hospital Clinico, Universidad de Chile: FWA00029089). The experimental workflow is provided in Fig. 1.

### Power calculations

A priori power calculations were conducted using G∗Power 3.1^42^ and indicated that a total sample size of 55 would be appropriate for our main behavioural analyses (e.g., linear regressions with 6 predictors) in each tandem group (effect size *f*^*2*^ = 0.15, power = 0.80, α = 0.05). Our samples exceeded this estimation in each tandem group and therefore our study was sufficiently powered. Moreover, our sample size is similar to other studies using EEG and VBM techniques in neurodegenerative conditions.^17^, ^18^, ^19^, ^20^

### Cognitive assessment

The Addenbrooke's Cognitive Examination-III (ACE-III)^43^ or Montreal Cognitive assessment (MoCA)^44^ were used to assess cognitive performance. Both ACE-III and MoCA provide measures of attention, memory, language, and visuospatial abilities. For comparison, ACE-III and MoCA were converted to Mini Mental State Examination (MMSE) based on previous conversion methods.^45^^,^^46^ Total MMSE-converted scores are out of 30, with higher scores representing greater cognitive performance.

### Executive function

The INECO Frontal screening battery (IFS)^47^ was used to assess frontal-executive function. The IFS measures motor programming and sequencing, inhibitory control, working memory, verbal fluency, abstract reasoning, and interference control. Total IFS scores are out of 30, with higher scores representing better executive function.

### Social cognition

The Mini-Social and Emotional Assessment (Mini-SEA)^48^^,^^49^ was used to assess social cognition. The Mini-SEA consists of two parts: i) facial emotion recognition test and ii) faux pas recognition test. The facial emotion recognition test uses Ekman's faces and requires participants to select an emotional label to match the presented face (i.e., fear, sadness, disgust, surprise, anger, and happiness). The faux pas recognition test measures theory of mind and is based on the ability to detect social faux pas based on short stories. Each part of the Mini-SEA is measured out of 15, with a total score of 30. Higher scores represent better social cognition performance.

### Interoception-exteroception task

A validated heartbeat detection (HBD) task was used,^18^, ^19^, ^20^^,^^32^, ^33^, ^34^^,^^50^ where participants completed two 2-min blocks of interoception or exteroception. Participants were required to tap a computer keyboard along with their heartbeat (interoception) or external audio stimuli (exteroception). Presentation order was counterbalanced. High density EEG signals were recorded during each condition (see EEG pre-processing). ECG was recorded via external electrodes.

### Neuroimaging acquisition

Whole-brain structural MRI data were obtained, and standard pre-processing steps were followed as recommended by the Organisation for Human Brain Mapping.^51^^,^^52^ Each centre followed standard protocols (Supplementary Table S3 for scanner details).^40^^,^^53^^,^^54^ Twenty-five participants (10 HCs, 7 bvFTD, and 8 AD) were excluded because of the absence of MRI recordings or artifacts (Supplementary Table S4 for demographic information of MRI sample).

### Statistics

#### Demographics

Demographic and neuropsychological variables were compared via independent t-tests (i.e., age, education, cognitive, and social cognition scores), or chi-square tests (i.e., biological sex - self-reported).

#### EEG pre-processing

High density EEG signals were recorded during each condition using a Biosemi Active-two 128-channel system at 1024 Hz. EEG data were resampled offline at 256 Hz and filtered at 0.5–30 μV to remove any unwanted frequency components (e.g., electrical interference, noise).^55^ A semi-automatic pipeline was used.^56^^,^^57^ Independent component analysis was used to correct eye movements, blink artifacts and cardiac field artifacts^58^ and verified using a visual inspection protocol.^18^^,^^20^^,^^59^ R-wave values from the ECG signal were identified with the pan Tompkins function on HEPLAB toolbox.^60^ The reference was set to linked mastoids for recording and re-referenced offline to the average of all the rest of the electrodes. Malfunctioning channels were replaced via statistically weighted spherical interpolation (based on neighbouring sensors).^61^ EEG data were split into continuous files for interoception or exteroception for each participant for ACW analyses described below.

#### Autocorrelation window (ACW)

The autocorrelation function (ACF) quantifies the correlation between a signal and a delayed version of itself at various time lags,^62^ indicative of the length of the intrinsic neuronal timescales.^63^, ^64^, ^65^, ^66^, ^67^ The ACW is defined as the time lag at which a specific value of autocorrelation is reached.^68^ In this work, we focused on the ACW-50, which marks the point at which the autocorrelation function intersects the correlation threshold of 0.5 and has been previously investigated in neuroimaging studies.^63^^,^^69^ We investigated ACW at the channel level by computing the ACW-50 on the broadband pre-processed signal, in line with previous works.^70^^,^^71^ The ACW, and the ACW-50 in particular, can be used as measures of the stability of a signal, where a random time series tends to exhibit an ACW-50 nearing zero, while a signal with slower dynamics manifests non-zero values. Using custom MATLAB code, we computed the ACF and ACW-50 using a predefined time lag of 1 sample (1/256 Hz = 0.004 s) for each heartbeat (t0) for each electrode and for each epoch (3-s windows) for both interoception and exteroception conditions. Then, we averaged over epochs to get a value per electrode which was used for the spatial clustering analysis. To ensure that the ACF always reached 0.5, in line with previous works,^15^^,^^35^^,^^70^^,^^71^ we also plotted the ACF function for each group (Supplementary Figure S1).

#### Spatiotemporal clustering analysis

To avoid *a priori* spatiotemporal bias and account for multiple comparisons, non-parametric data driven spatiotemporal clustering was performed.^72^, ^73^, ^74^ ACW differences between groups were assessed in interoception and exteroception through cluster-based topographic analyses. First, for each between-group comparison, we performed a Wilcoxon test—a univariate non-parametric test that does not assume normal distribution^75^—on the ACW values associated with each electrode and obtained the associated *p*-values for each electrode. Next, we set a conservative threshold of *p* < 0.025 to define clusters of neighbouring electrodes to identify potential differences between groups. To be considered significant, clusters needed to encompass 5 or more electrodes.^73^ To assess the significance of the identified spatiotemporal clusters, permutation-based testing was used (5000 permutations), with recombination and randomised resampling of the participant-wise averages before each repetition using a Monte Carlo method. The cluster-level statistic was set to *p* < 0.05.

#### Heart-evoked potential

To examine the HEP in interoception and exteroception, we segmented the continuous EEG signal into epochs from −300 to 600 ms around the R-wave peak in each condition and baseline-corrected relative to the −300 ms time window preceding the heartbeat.^17^^,^^20^^,^^59^ Low drifts were removed by linear trend corrections.^55^ Next, noisy epochs were rejected when trials exceed a threshold of 2.5 standard deviations from the mean probability distribution calculated from all trials and using probability distribution kurtosis.^76^ Trials were averaged across subjects for group comparisons between conditions (Supplementary Figure S2a and b).

#### Predictors of behavioural measures

Multiple linear regressions were run to predict the following behavioural outcomes: i) global cognition; ii) executive function; iii) overall social cognition; and iv) emotion recognition. Each model included the following predictors: i) dummy coded diagnosis (bvFTD-control tandem; or AD-control tandem); ii) ACW-50 average cluster value for relevant cluster; iii) age; iv) biological sex - self-reported (male or female); v) education; and vi) site. These predictors were chosen based on previous evidence reporting a relationship between interoception and sociocognitive measures,^17^^,^^19^^,^^20^^,^^32^ while controlling for the influence of demographic variables. All *p*-values for predictors were FDR-corrected using the Benjamini-Hochberg method.^77^ Further, we repeated our multiple regression models including the HEP (average modulation between 200 and 500 ms) to investigate whether the pattern of results was influenced by the HEP. All behavioural analyses were conducted using Python (v.3.10.12) with Pandas package (v.2.0.3)^78^ and Statsmodels package (v.0.14.2).^79^ Assumptions of linear regression (e.g., linearity, normality, and multicollinearity) were assessed (Supplementary Materials). The normality assumption was violated in several models and transformations did not improve normality. For consistency, all regression models were conducted with bootstrapping techniques with 5000 iterations to increase precision.^80^ Bootstrapped coefficients and 95% CI are reported alongside the original models for comparison. Missing data was imputed using an iterative imputer with a Bayesian ridge model as the estimator using Sci-kit learn package (v.1.6).^81^ Comparisons of missing and raw data are reported in Supplementary Tables S5 and S6.

#### Relationship with interoceptive accuracy, heart rate variability and HEP

Pearson's correlations were conducted to investigate the relationship between the ACW-50 cluster and interoceptive accuracy, as measured by the mean distance index^82^ and heartrate variability (RR-interval and SD of the RR interval),^83^ and the HEP (average modulation between 200 and 500 ms). Group differences in interoceptive accuracy, heartrate variability, and HEP are reported in Supplementary Materials (Supplementary Table S9, Supplementary Figure S2a and b).

#### MRI pre-processing and analysis

Voxel-based morphometry (VBM) was performed using the Computational Anatomy Toolbox (CAT12, https://neuro-jena.github.io/cat/) in MATLAB R2022a. Standard pre-processing steps included bias-field correction, noise reduction, skull stripping, segmentation, and normalisation to the Montreal Neurological Institute (MNI) space with a resolution of 1.5 mm isotropic, using default parameters. Sample homogeneity and orthogonality checks were performed. Regions of interest (ROI) masks were created using the MarsBar toolbox^84^ for the AIN-core (bilateral insula, anterior cingulate cortex, and amygdala) and AIN-extended regions (bilateral insula, anterior cingulate cortex, and amygdala, together with the bilateral orbitofrontal cortex, mid cingulate cortex, angular gyrus, hippocampus, thalamus, and ventral striatum)^2^^,^^3^ using the Automated Anatomical Labelling (AAL-3) atlas.^85^ Within ROI masks, regression analyses were conducted with the ACW-50, controlling for group and scanner. All clusters were FDR-corrected, *p* < 0.05, with a cluster extent threshold of 50 contiguous voxels.

### Role of funders

Funding bodies played no direct role in study design, data collection, analysis, or interpretation of data, manuscript preparation or decision to submit for publication.

## Results

### Demographics, cognitive, and social-cognitive performance

Demographic information is shown in Table 1. No differences were observed in age, sex, or education between patient and HC tandem groups. Both patient groups showed worse cognitive scores, executive dysfunction, and social cognition impairment relative to controls.

**Table 1:** Demographic, cognitive and social-cognitive assessments in AD, bvFTD and healthy controls.

	Group		Statistic	*p*
	HC	AD		
	n = 34	n = 35		
Age	72.44 ± 5.58	73.37 ± 7.65	−0.58	0.58
Sex (M:F)	13:21	12:23	0.116	0.73
Education (years)	13.32 ± 3.57	12.49 ± 4.91	0.81	0.42
MMSE-converted (/30)	28.03 ± 1.14	22.74 ± 2.85	10.16	<0.001
IFS (/30)	22.24 ± 3.04	15.05 ± 4.92	7.09	<0.001
Mini-SEA total (/30)	24.90 ± 2.60	19.21 ± 3.68	5.41	<0.001
Mini-SEA emotion (/15)	12.04 ± 1.54	10.47 ± 2.19	2.92	0.003
Mini-SEA faux pas (/15)	12.93 ± 1.87	9.38 ± 2.44	4.98	<0.001

	HC	bvFTD		
	n = 31	n = 31		
Age	66.94 ± 9.06	67.87 ± 11.38	−0.35	0.73
Sex (M:F)	18:13	15:16	0.58	0.45
Education (years)	15.06 ± 3.55	14.48 ± 4.82	0.53	0.60
MMSE-converted (/30)	28.04 ± 1.17	25.24 ± 2.64	5.19	<0.001
IFS (/30)	23.01 ± 3.78	18.11 ± 4.98	4.29	<0.001
Mini-SEA total (/30)	24.54 ± 2.20	20.47 ± 5.80	3.29	0.001
Mini-SEA emotion (/15)	11.79 ± 1.35	10.16 ± 2.64	2.71	0.01
Mini-SEA faux pas (/15)	13.01 ± 1.88	11.53 ± 2.25	2.29	0.01

Note. Values represent mean ± standard deviation unless otherwise specified. Statistics represent independent samples t-tests, except for Sex, where chi-square statistics are reported. Abbreviations: AD, Alzheimer's Disease; bvFTD, behavioural variant frontotemporal dementia; HC, Healthy controls.

### Spatiotemporal cluster analysis

#### ACW-50

##### bvFTD

**Fig. 2:**
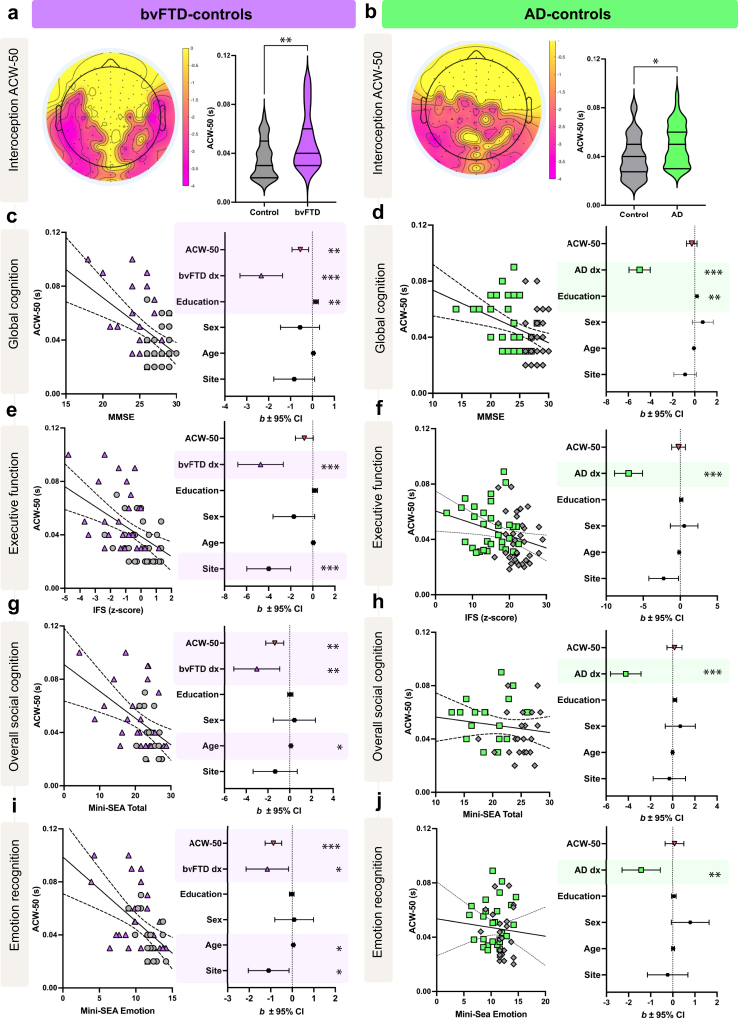
**Spatiotemporal dynamics of interoception in patients compared with controls.** ACW-50 cluster differences between a) bvFTD and controls, b) AD and controls for interoception. No significant differences were observed during exteroception. Regression models between ACW-50 average cluster scores for interoception are shown for global cognition in c) bvFTD patients and controls, and d) for AD patients and controls; for executive function in e) bvFTD patients and controls, and f) AD patients and controls; for overall social cognition in g) bvFTD patients and controls and h) AD patients and controls; and for emotion recognition in i) bvFTD patients and controls, and j) AD patients and controls. Significant predictors within each model are highlighted in purple (bvFTD) and green (AD). All predictors were FDR corrected, ∗*p* < 0.05, ∗∗*p* < 0.01, ∗∗∗*p* < 0.001. Sample size for bvFTD-control tandem: bvFTD, n = 31; CN, n = 31. Sample size for AD-control tandem: AD n = 35, CN, n = 34; Abbreviations: ACW, Autocorrelation window; AD, Alzheimer's disease; bvFTD, behavioural variant frontotemporal dementia; IFS, INECO frontal screening battery; Mini-SEA, Mini Social Emotional Assessment; MMSE, Mini Mental State Exam; s, seconds.

During interoception, the ACW-50 differed significantly between bvFTD and HC in the bilateral frontotemporal and parietal regions (Fig. 2a). No significant differences were observed in exteroception between groups.

##### AD

During interoception, the ACW-50 differed significantly between AD and HC in the central-parietal regions (Fig. 2b). No significant differences were observed in exteroception between groups.

#### Predictors of cognition, executive dysfunction, and social cognition

We conducted multiple linear regressions to investigate if the ACW-50 average cluster from each patient group during interoception (bvFTD-controls, Fig. 2c, e, g, i; and AD-controls, Fig. 2d, f, h, g) predicted cognitive and socioemotional measures (Tables 2 and 3).

**Table 2:** Predictors of cognitive performance.

	Cognition					Executive functioning				
bvFTD v controls										
Overall model	*F* (6, 55) = 10.99, *p* < 0.001, *R*^*2*^ = 0.545					*F* (6, 55) = 9.49, *p* < 0.001, *R*^*2*^ = 0.509				
	b(SE)	*t*	*p*	b(SE)∗	95% CI [L, U]∗	b(SE)	*t*	*p*	b(SE)∗	95% CI [L, U]∗
Constant	24.79 (2.16)	11.48	<0.001	24.78 (2.25)	[20.13, 28.99]	24.47 (4.58)	5.57	<0.001	25.47 (5.00)	[16.33, 35.77]
Age	0.04 (0.02)	1.84	0.093	0.04 (0.02)	[−0.01, 0.08]	0.05 (0.05)	0.96	0.344	0.05 (0.06)	[−0.09, 0.16]
Sex	−0.56 (0.45)	−1.27	0.209	−0.57 (0.46)	[−1.46, 0.34]	−1.72 (0.94)	−1.82	0.103	−1.72 (0.92)	[−3.46, 0.11]
Education	0.16 (0.06)	2.99	0.009∗∗	0.16 (0.06)	[0.05, 0.28]	0.19 (0.12)	1.66	0.119	0.19 (0.12)	[−0.06, 0.40]
Site	−0.83 (0.47)	−1.78	0.093	−0.83 (0.49)	[−1.82, 0.10]	−4.00 (0.99)	−4.04	<0.001∗∗∗	−4.00 (1.02)	[−6.09, −2.06]
Diagnosis	−2.34 (0.49)	−4.82	<0.001∗∗∗	−2.34 (0.42)	[−3.14, −1.49]	−4.74 (1.03)	−4.61	<0.001∗∗∗	−4.74 (1.00)	[−6.74, −2.84]
ACW-50	−0.55 (0.19)	−2.91	0.009∗∗	−0.55 (0.25)	[−1.06, −0.08]	−0.77 (0.40)	−1.91	0.103	−0.77 (0.44)	[−1.62, 0.08]

AD v controls										
Overall model	*F* (6, 62) = 28.78, *p* < 0.001, *R*^*2*^ = 0.732					*F* (6, 62) = 14.14, *p* < 0.001, *R*^*2*^ = 0.578				
	b(SE)	*t*	*p*	b(SE)∗	95% CI [L, U]∗	b(SE)	*t*	*p*	b(SE)∗	95% CI [L, U]∗
Constant	30.98 (2.75)	11.29	<0.001	30.98 (2.79)	[25.04, 35.95]	34.73 (5.41)	6.42	<0.001	34.73 (4.36)	[26.06, 43.81]
Age	−0.07 (0.04)	−2.04	0.090	−0.07 (0.04)	[−0.14, 0.01]	−0.16 (0.07)	−2.15	0.072	−0.16 (0.06)	[−0.28, −0.04]
Sex	0.72 (0.48)	1.12	0.161	0.72 (0.47)	[−0.15, 1.69]	0.55 (0.97)	0.57	0.571	0.52 (0.94)	[−1.21, 2.48]
Education	0.20 (0.06)	3.40	0.003∗∗	0.20 (0.06)	[0.08, 0.32]	0.14 (0.12)	1.14	0.387	0.13 (0.12)	[−0.12, 0.35]
Site	−0.87 (0.51)	−1.71	0.138	−0.87 (0.48)	[−1.77, 0.11]	−2.31 (1.03)	−2.24	0.072	−2.25 (1.00)	[−4.08, −0.18]
Diagnosis	−4.98 (0.48)	−10.39	0.003∗∗	−4.98 (0.46)	[−5.97, −4.15]	−7.08 (0.97)	−7.27	0.006∗∗	−6.99 (0.97)	[−8.91, −5.08]
ACW-50	−0.27 (0.24)	−1.14	0.260	−0.27 (0.20)	[−0.71, 0.10]	−0.31 (0.48)	−0.64	0.571	−0.23 (0.44)	[−1.13, 0.60]

Note. All *p* values are FDR-corrected. ∗*p* < 0.05; ∗∗*p* < 0.01; ∗∗∗*p* < 0.001. b(SE)∗ represent bootstrapped coefficient and standard error values and 95% CI [L, U]∗ represent bootstrapped confidence intervals. All bootstrapping was performed using 5000 iterations. Abbreviations: L, Lower CI; U, Upper CI.

**Table 3:** Predictors of social cognition and emotion recognition performance.

	Social cognition^a^					Emotion recognition^a^				
bvFTD v controls										
Overall model	*F* (6, 55) = 5.89, *p* < 0.001, *R*^*2*^ = 0.391					*F* (6, 55) = 8.30, *p* < 0.001, *R*^*2*^ = 0.475				
	b(SE)	*t*	*p*	b(SE)∗	95% CI [L, U]∗	b(SE)	*t*	*p*	b(SE)∗	95% CI [L, U]∗
Constant	17.97 (4.70)	3.83	0.002	17.98 (4.32)	[9.49, 26.75]	10.89 (2.18)	4.63	<0.001	10.09 (2.51)	[5.59, 15.65]
Age	0.11 (0.05)	2.13	0.066	0.11 (0.05)	[−0.01, 0.19]	0.06 (0.02)	2.38	0.030∗	0.06 (0.03)	[−0.01, 0.10]
Sex	0.43 (0.97)	0.45	0.657	0.43 (0.98)	[−1.47, 2.39]	0.09 (0.45)	0.20	0.841	0.09 (0.46)	[−0.81, 0.98]
Education	0.05 (0.12)	0.45	0.657	0.05 (0.15)	[−0.24, 0.34]	−0.02 (0.06)	−0.42	0.789	−0.02 (0.06)	[−0.14, 0.09]
Site	−1.34 (1.01)	−1.32	0.271	−1.36 (0.94)	[−3.07, 0.59]	−1.09 (0.47)	−2.32	0.033∗	−1.09 (0.45)	[−1.99, −0.22]
Diagnosis	−3.03 (1.06)	−2.87	0.014∗	−3.03 (0.88)	[−4.79, −1.31]	−1.15 (0.49)	−2.36	0.033∗	−1.15 (0.51)	[−2.12, −0.12]
ACW-50	−1.38 (0.41)	−3.34	0.005∗∗	−1.38 (0.52)	[−2.50, −0.47]	−0.87 (0.19)	−4.55	<0.001∗∗∗	−0.87 (0.22)	[−1.30, −0.46]

Overall model	*F* (6, 62) = 8.95, *p* < 0.001, *R*^*2*^ = 0.464					*F* (6, 62) = 2.87, *p* < 0.016, *R*^*2*^ = 0.218				
	b(SE)	*t*	*p*	b(SE)∗	95% CI [L, U]∗	b(SE)	*t*	*p*	b(SE)∗	95% CI [L, U]∗
Constant	22.13 (3.94)	5.61	<0.001	22.13 (4.41)	[11.14, 28.59]	9.20 (2.48)	3.71	<0.001	9.20 (2.94)	[2.64, 14.21]
Age	−0.01 (0.05)	−0.22	0.824	−0.01 (0.06)	[−0.11, 0.14]	0.01 (0.03)	0.44	0.661	0.01 (0.04)	[−0.06, 0.11]
Sex	0.69 (0.68)	1.01	0.632	0.69 (0.66)	[−0.58, 1.95]	0.79 (0.43)	1.85	0.207	0.79 (0.42)	[−0.03, 1.63]
Education	0.20 (0.08)	2.41	0.057	0.20 (0.06)	[0.07, 0.32]	0.05 (0.05)	0.90	0.717	0.05 (0.04)	[−0.04, 0.13]
Site	−0.30 (0.73)	−0.42	0.814	−0.30 (0.66)	[−1.59, 1.01]	−0.23 (0.46)	−0.50	0.717	−0.23 (0.42)	[−1.04, 0.60]
Diagnosis	−4.23 (0.69)	−6.15	0.006∗∗	−4.23 (0.67)	[−5.59, −2.91]	−1.42 (0.43)	−3.28	0.012∗	−1.42 (0.42)	[−2.26, −0.62]
ACW-50	0.16 (0.34)	0.48	0.814	0.16 (0.31)	[−0.48, 0.73]	0.07 (0.21)	0.36	0.717	0.08 (0.18)	[−0.28, 0.45]

Note. All *p* values are FDR-corrected. ∗*p* < 0.05; ∗∗*p* < 0.01; ∗∗∗*p* < 0.001. b(SE)∗ represent bootstrapped coefficient and standard error values and 95% CI [L, U]∗ represent bootstrapped confidence intervals. All bootstrapping was performed using 5000 iterations. Abbreviations: L, Lower CI; U, Upper CI.

a Based on imputed values to handle missing data, raw regression models reported in Supplementary Tables S3 and S4 for comparison.

##### bvFTD-controls

Worse cognition was predicted by longer ACW-50 cluster values during interoception, together with a diagnosis of bvFTD, and less education (Table 2, Fig. 2c). Worse overall social cognition was predicted by interoceptive longer ACW-50, together with a diagnosis of bvFTD (Table 3, Fig. 2g). Worse emotion recognition was predicted by interoceptive longer ACW-50, with a trend for diagnosis of bvFTD observed (Table 3, Fig. 2i). Worse executive functioning was predicted by diagnosis of bvFTD and site (Table 2, Fig. 2e).

##### AD-controls

Worse cognition, executive function, social cognition, and emotion recognition were predicted by a diagnosis of AD, with less education also predicting worse cognition (Tables 2 and 3, Fig. 2d, f, h, g). ACW-50 was not a significant predictor of any behavioural outcome in AD.

All regression results in bvFTD and AD patients remained unchanged when accounting for HEP modulation (Supplementary Tables S7 and S8).

### Relationship between ACW-50 clusters, interoceptive accuracy, and heart rate variability, and HEP

Both patient groups showed reduced HEP modulation in the interoceptive in comparison with exteroceptive condition (Supplementary Figure S2a), both only bvFTD showed a selective interoceptive deficit when compared with controls (Supplementary Figure S2b). Further, both patient groups showed worse interoceptive accuracy than controls. No differences were observed in heart-rate variability (R-R interval in ms). No associations were observed between ACW-50 clusters and interoceptive accuracy, heart-rate variability metrics, or average HEP in the bvFTD-control or AD-control groups (all *p*'s > 0.10) (Supplementary Material).

### Neuroimaging results

#### Associations with ACW-50

##### bvFTD-controls

**Fig. 3:**
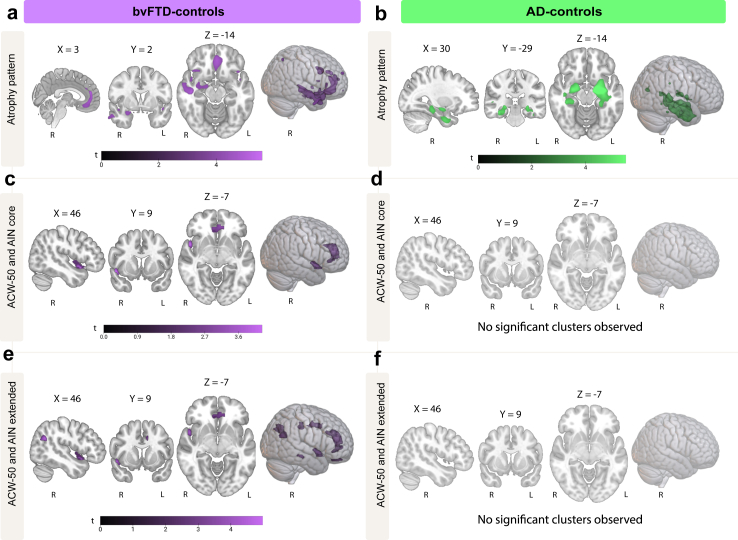
**Structural based associations within the allostatic interoceptive network.** Voxel-based morphometry showing patterns of atrophy in bvFTD a) and AD patients b). Reduced structural integrity was associated with longer ACW-50 in AIN core regions in bvFTD patients c) but not in AD patients d). Reduced structural integrity was associated with longer ACW-50 in AIN extended regions in bvFTD patients e), but not in AD patients f). All imaging analyses included diagnosis and site as nuisance variables. MNI coordinates are displayed above brain slices. Colour bars represent *t*-values. All clusters reported at FDR *p* < 0.05, corrected for multiple comparisons. Sample size A1-A3: bvFTD n = 24, CN n = 21; Sample size B1–B3: AD = 27, CN = 24; Abbreviations: R, Right; L, Left. Brain slices displayed in radiological orientation.

In bvFTD, longer interoceptive ACW-50 values in the bilateral frontotemporal and parietal clusters (Fig. 2a) were associated with reduced structural integrity of the right insula and bilateral pregenual and superior ACC within the AIN-core mask (all FDR-corrected *p* < 0.05, Fig. 3c, Table 4), together with the bilateral angular gyrus, bilateral MCC, right hippocampus, and bilateral orbitofrontal cortex within the AIN-extended mask (all FDR-corrected *p* < 0.05, Fig. 3e, Table 4).

**Table 4:** Structural neural correlates of ACW-50 in bvFTD.

Region	MNI						
	Side	Size	X	Y	Z	*t*	FDR *p*
**AIN - core**							
Insula	R	290	48	9	−11	4.31	0.007
Pregenual ACC and superior ACC	R	2255	8	45	15	4.25	0.007
Pregenual ACC	Bi	–	14	39	15	4.18	0.007
Pregenual ACC	R	–	6	39	26	3.94	0.008
**AIN - extended**							
Angular gyrus	R	447	50	−57	24	5.02	0.016
Angular gyrus	L	653	−47	−68	26	4.63	0.016
Angular gyrus	L	–	−39	−71	35	3.58	0.019
Angular gyrus	L	–	−47	−80	90	3.47	0.016
Insula	R	238	48	9	−11	4.30	0.016
Pregenual ACC and superior ACC	R	1490	8	45	15	4.23	0.016
Pregenual ACC	Bi	–	14	39	15	4.14	0.016
Pregenual ACC	R	–	6	39	26	3.92	0.016
Hippocampus	R	72	41	−29	−15	3.58	0.019
MCC	L	389	−12	3	44	3.58	0.019
MCC	L	–	−9	−8	39	3.30	0.022
MCC	L	–	−11	14	44	2.75	0.036
MCC	R	213	9	−26	38	3.55	0.016
MCC	R	–	17	−27	48	2.79	0.035
Pregenual ACC	L	500	−8	41	3	3.46	0.019
Superior ACC	Bi	–	−11	36	−9	3.09	0.024
Medial OFC	R	–	5	38	−8	2.94	0.032
Medial OFC	L	95	−8	44	−27	2.95	0.031

Note. All clusters reported at FDR *p* < 0.05, corrected for multiple comparisons. Abbreviations: ACC, Anterior Cingulate Cortex; MCC, Midcingulate cortex; OFC, Orbitofrontal cortex; R, right; L, Left; Bi, Bilateral.

##### AD-controls

In AD, no correlations between interoceptive ACW-50 and ROI regions survived statistical threshold corrections (all FDR-corrected *p* > 0.05, Fig. 3b, d, f).

## Discussion

Our study provides empirical evidence of altered spatiotemporal brain dynamics associated with interoception in bvFTD and AD. Altered spatiotemporal brain dynamics of interoception were related to impaired sociocognitive functioning in bvFTD only, suggesting a core allostatic-interoceptive deficit in this condition. Neuroimaging analyses uncovered associations between altered spatiotemporal brain dynamics and structural integrity of key areas of the allostatic-interoceptive network, highlighting the neurobiological mechanism of this deficit in bvFTD. This evidence suggests that dysfunctional intrinsic neural timescales of interoception could be a mechanism for the neurocognitive impairments observed in patients with bvFTD. In the following sections, we consider how our results are in line with the recently proposed PAIO-INT theoretical framework^11^ and how these measures may inform clinical understanding of these diseases.

Our findings in bvFTD support the presence of a core allostatic-interoceptive deficit.^14^ Behavioural, physiological, and neuroimaging measures from previous studies support similar conclusions.^17^, ^18^, ^19^, ^20^ Our findings extend previous research by uncovering altered intrinsic timescales (longer ACW during interoception but not exteroception) in bvFTD and its specific association with neurocognitive core deficits. While deficits in the HEP were observed in both patient groups, a selective impairment was observed in bvFTD during the interoceptive condition, in line with previous reports.^17^^,^^18^^,^^20^ However, the dynamic measures of intrinsic neural timescales during interoception were not explained by static measures, such as the HEP modulation or HRV. Slower intrinsic timescales during interoception may result in dysfunctions in the brain's predictive coding capacities.^11^ Altered intrinsic neural timescales of interoception were linked to key AIN structures and relevant sociocognitive measures in bvFTD. This evidence supports the idea that the maladaptive environmental responses in this disease are due to a core allostatic-interoceptive deficit,^11^^,^^14^ and may be due to failures of temporal segregation and integration of relevant internal and external signals.^11^ Altered intrinsic neural timescales of interoception could represent an early marker of brain disease, prior to the onset of observable clinical symptoms in bvFTD. Indeed, the regions of the AIN, such as the insula and anterior cingulate cortex, are vulnerable to early pathophysiological changes in bvFTD^24^ and have been reported up to 10 years before disease onset.^86^^,^^87^ Our work opens avenues for future research to consider the role of early allostatic-interoceptive deficits in bvFTD as a marker of disease. Importantly, our findings complement and extend upon static measures of interoception and anatomical structure-function brain mapping by uncovering dynamic spatiotemporal hierarchies to further our understanding of brain health and disease.^11^

Altered timescales of interoception appeared to also be relevant to AD. This may relate to a generalised disruption of brain oscillations in AD, further supported by the non-specific reduced modulation of the HEP for interoception and exteroception observed in our study. Emerging evidence has reported that in AD, the disruption of the brain's intrinsic temporal irreversibility (i.e., the temporal asymmetry of brain dynamics) occurs across the whole brain and with all frequency bands.^29^ In our study, however, the longer timescales during interoception in AD had no relationship to clinical features or neuroanatomical correlates of the disease. This is not surprising, as previous research in AD in interoception has also produced mixed findings at the behavioural and neural level,^18^, ^19^, ^20^ in comparison with bvFTD where allostatic-interoceptive deficits appear to be pervasive.^11^^,^^14^^,^^16^, ^17^, ^18^, ^19^, ^20^ This different pattern of results in bvFTD and AD may reflect the preferential and widespread damage within the AIN in bvFTD^11^^,^^14^^,^^16^, ^17^, ^18^, ^19^, ^20^^,^^24^ whereas in AD, AIN damage appears to be more circumscribed.^17^^,^^38^^,^^88^ Taken together, our results suggest that temporal dynamics of interoception in AD are not a *sui generis* deficit, with no specific anatomical and behavioural associations.

Our findings complement evidence from psychiatric populations,^35^^,^^89^^,^^90^ suggesting that dysfunctional allostatic-interoception may be a transdiagnostic feature.^11^ Meta-analytic evidence has shown that disruptions in AIN occur in both bvFTD and psychiatric conditions.^91^^,^^92^ Moreover, a substantial overlap in symptoms exists between bvFTD and psychiatric conditions, leading to diagnostic delays due to common features.^93^ Altered intrinsic neural timescales measured by the ACW are observed in psychiatric conditions during resting state and self-referential processing.^35^^,^^89^^,^^90^ To our knowledge, altered neural timescales related to interoception have not been investigated in psychiatric conditions alone or with neurodegenerative diseases, and represents an opportunity to further understand the transdiagnostic applications.^11^ Such investigations will further refine our growing synergistic understanding of brain health and disease.

The current study had some limitations. First, direct comparisons were not made between AD and bvFTD due to differences in sample characteristics (e.g., demographics). Therefore, it is currently unknown whether differences in spatiotemporal brain dynamics of interoception between dementia syndromes exist. Second, patients received diagnoses based on established clinical criteria only,^37^^,^^38^ without testing for ATN biomarkers in AD such as the deposition of amyloid-β and tau proteins established via Positron Emission Tomography, CSF, or plasma in AD.^94^, ^95^, ^96^, ^97^ Further, biomarkers relevant for bvFTD, such as neurofilament light chain in CSF and synaptic proteins such as Synaptophysin and GAP43^98^, ^99^, ^100^ were not available. The current clinical diagnostic criteria, however, is the current gold standard for clinical diagnosis within the literature and has been used globally to diagnose AD and bvFTD.^53^^,^^56^^,^^101^^,^^102^ Moreover, another challenge is the accessibility and feasibility of these biomarkers in global settings, together with the current lack of systematic validation of these biomarkers in diverse populations,^103^ such as the cohort used in the current paper. Future works should seek to incorporate clinical and biomarker criteria to model spatiotemporal brain dynamics to further our understanding in these disease groups. Next, we focused on the broadband EEG signal, as no previous work has investigated INT in people with bvFTD or AD. While this approach has also been followed in other clinical populations, such a schizophrenia,^35^ whether the interoceptive INTs differ across frequency bands in neurodegeneration warrants future investigation. Altered brain oscillations are observed in neurodegeneration, such as in alpha and beta bands in AD and alpha and gamma bands in bvFTD.^104^ Emerging work has reported that longer resting-state INTs were associated with lower alpha peak frequencies in different states of consciousness.^70^ How this relationship may function during interoception in neurodegeneration remains an open avenue for investigation. Additionally, in the current work we focused on the time-domain of INT,^15^ in line with previous works.^35^^,^^70^ Other measures in the frequency-domain exist, such as the power law exponent (PLE),^15^ which has been related to spectral entropy.^105^ It is possible that other measures of INT such as PLE and measures of spectral entropy are also altered during neurodegenerative processes and warrants future investigation. Next, we focused here on cardiac interoceptive inputs. This limitation is not unique to our study and also influences a majority of interoception research (for meta-analysis see^106^). Further research across multiple interoceptive inputs is required to gain a deeper understanding of how the brain processes multiple interoceptive inputs, governed by different frequencies and timescales under the PAIO-INT framework. Finally, in our sample, we investigated structural associations with ACW, using the AAL3 parcellation. Although the AAL3 has been systematically used in neurodegenerative research^101^^,^^107^, ^108^, ^109^ and yields similar results when compared to other parcellation methods,^101^ it is purely anatomical (i.e., does not provide functional information). Further research investigating the ACW with functional networks within the AIN is needed and represents an opportunity for future research in this field.

Cognitive neuroscience is moving towards a synergistic understanding of the continuum between brain health and disease that considers the complex interplay between the brain, body, and environment.^11^^,^^12^ Our study provides evidence of altered intrinsic neural timescales during interoception in neurodegenerative diseases, with relevance for bvFTD. Moreover, our findings support that altered intrinsic timescales represent a plausible neurobiological mechanism underpinning the anatomical and behavioural changes observed within this syndrome. Our study paves the way for future research to consider altered intrinsic neural timescales of interoception as an early marker of disease, as well as transdiagnostic investigations spanning neurological and psychiatric conditions.

## Contributors

**JLH**, **GDB**, and **AI** accessed and verified the underlying data supporting the findings in this manuscript.

**JLH:** Conceptualisation; Methodology; Formal analysis; Writing – Original Draft; Visualisation; **GDB:** Methodology; Software; Formal analysis; Validation; Writing – Original Draft; Visualisation; **PB:** Software; Data curation; Writing – review and editing; **MD:** Software; Writing – review and editing; **RGG:** Data curation; Writing – review and editing; **JM:** Writing – review and editing; **SM:** Formal analysis; Writing – review and editing; **AL:** Investigation; Software, Writing – review and editing; **HH:** Formal analysis; Writing – review and editing; **PP:** Formal analysis; Writing – review and editing; **JC:** Formal analysis; Writing – review and editing; **MM:** Software; Writing – review and editing; **MF-V:** Investigation; Software; Writing – review and editing; **MLGG:** Software; Writing – review and editing; **YC:** Software; Writing – review and editing; **BM:** Writing – review and editing; **OP:** Writing – review and editing; **GN:** Conceptualisation; Writing – review and editing; **AI:** Conceptualisation; Methodology; Resources; Project administration; Funding acquisition; Writing – Original Draft. All authors read and approved the final version of this manuscript.

## Data sharing statement

Anonymised data that support the study findings are drawn from the BrainLat project,^36^^,^^110^ a large open access multimodal neuroimaging database that can be found here: https://www.synapse.org/Synapse:syn51549340/wiki/624187.^111^ Supporting code can be found here: https://osf.io/h9pse/.

## Declaration of interests

OP holds an unpaid position as the President (2025–2026) of the International Society for Frontotemporal Dementias. BM holds royalties and/or licences with Cambridge University Press, Elsevier, Inc., Guildford Publications, Inc., Johns Hopkins Press, Oxford University Press, Taylor & Francis Group. BM was paid consulting fees by Massachusetts General Hospital Alzheimer's Disease Research Centre (ADRC) Scientific Advisory Board (SAB) (2021, 2022, and 2023), by Stanford University ADRC SAB (2021, 2022, and 2023), by University of Washington ADRC SAB (2021, 2022, and 2023), and Genworth Medical Advisory Board (March 2023). BM received payment or honoraria for the following lectures/presentations/speakers' bureaus, manuscript writing or educational events by Fromm Institute for Lifelong Learning (May 2023), Global Summit on Neurodegenerative Diseases (Jun 2021), Korean Dementia Society (Jul 2022), Massachusetts General Hospital dementia course (2022, 2023), National MS society – Don Paty Lectureship (Jun 2021), Ochsner Neuroscience Institute (Nov 2021), Providence Saint Joseph Medical Center (Sep 2021), Taipei Medical University, Dementia Center (Mar 2022), UC Irvine Institute for Memory Impairments and Neurological Disorders (UCI MIND) (Mar 2022), University of California, Los Angeles (UCLA) Grand Rounds (Apr 2022), University of Texas, Center for Brain Health (Jan 2021). BM received support for attending meetings and/or travel to the Association for Frontotemporal Degeneration (AFTD) Education Symposium, St. Louis, MO (May 2023), Milken Institute FTD Scientific Retreat, Los Angeles, CA (Mar 2023), California Institute of the Arts, Los Angeles, CA (Apr 2022), UCLA (Apr 2022). BM Participates on the following data safety monitoring board/advisory boards: Arizona Alzheimer's Consortium (External Advisor), Association for Frontotemporal Degeneration (Scientific Advisor), The Buck Institute for Research on Aging (Scientific Advisor), Cure ALE (Scientific Advisor), The John Douglas French Alzheimer's Foundation (Medical Advisor), Fundación Centro de Investigación Enfermedades Neurológicas, Madrid, Spain (Scientific Advisor), Genworth (Scientific Advisor), Kissick Family Foundation (Scientific Advisor), The Larry L. Hillblom Foundation (Scientific Advisor), Massachusetts General Hospital ADRC (Scientific Advisor), National Institute for Health Research Cambridge Biomedical Research Center and its subunit, the Biomedical Research Unit in Dementia (Scientific Advisor), Stanford University ADRC Scientific Advisor), University of Southern California P01 Urban Air Pollution and Alzheimer's Disease: Risk, Heterogeneity, and Mechanisms (External Advisory Committee), University of Washington ADRC (Scientific Advisor). BM holds the leadership or fiduciary roles in the following board/society/committees: The Bluefield Project to Cure FTD (Director and Internal Advisor), Global Brain Health Institute (Founding Director), Institute for Neurodegenerative Diseases (Affiliated Faculty), and Tau Consortium of the Rainwater Charitable Foundation (Co-Director and Scientific Advisor).
