# Supplementary material for: Altered spatiotemporal brain dynamics of interoception in behavioural-variant frontotemporal dementia
Source: eBioMedicine. 2025 Feb 22;113:105614. doi: 10.1016/j.ebiom.2025.105614 (PMC11894334; doi:10.1016/j.ebiom.2025.105614)
Supplement: Supplementary Materials [file mmc1.docx]

**Title: Altered spatiotemporal brain dynamics of interoception in behavioral-variant frontotemporal dementia**

**Authors:** Jessica L. Hazelton^†,1,2,3^, Gabriel Della Bella^†,4,5^, Pablo Barttfeld^4^, Martin Dottori^2^, Raul Gonzalez-Gomez^1^, Joaquín Migeot^1^, Sebastian Moguilner^1^, Agustina Legaz^1,2^, Hernan Hernandez^1^, Pavel Prado^6^, Jhosmary Cuadros^1,7,8^, Marcelo Maito^1,2^, Matias Fraile-Vazquez^1,2,9^, María Luz González Gadea^1,10^, Yasir Çatal^11^, Bruce Miller^12^, Olivier Piguet^3^, Georg Northoff^11,13,14^, Agustin Ibáñez^,1,2,12^

^†^These authors contributed equally to this work.

**Supplementary materials**

Supplementary Table 1. Atrophy patterns in bvFTD vs controls.

| Region | Side | MNI | | | Cluster | Voxels | *t* |
| --- | --- | --- | --- | --- | --- | --- | --- |
|  |  | X | Y | Z |  |  |  |
| Anterior cingulate cortex, paracingulate cortex, frontal pole, medial prefrontal cortex, subcallosal cortex | Bi | 0 | 46 | 10 | 1 | 9576 | 5.63 |
| Paracingulate cortex | Bi | 0 | 42 | -6 | 1 | - | 5.28 |
| Orbitofrontal cortex, temporal pole insula, frontal operculum cortex, central operculum cortex | L | -32 | 12 | -22 | 1 | - | 4.68 |
| Middle frontal gyrus, inferior frontal gyrus (pars triangularis, pars opercularis), precentral gyrus | L | -44 | 20 | 26 | 2 | 1084 | 5.63 |
| Middle frontal gyrus, inferior frontal gyrus (pars opercularis), precentral gyrus | L | -48 | 12 | 30 | 2 | - | 4.53 |
| Inferior frontal gyrus (pars triangualris), frontal pole, and middle frontal gyrus | L | -44 | 34 | 15 | 2 | - | 3.4 |
| Temporal pole, insula, middle temporal gyrus, inferior temporal gyrus superior temporal gyrus, inferior frontal gyrus (pars triangularis), frontal pole, frontal operculum cortex, orbitofrontal cortex | R | 46 | 8 | -30 | 3 | 14925 | 5.41 |
| Middle temporal gyrus, superior temporal gyrus, hippocampus, parahippocampal gyrus, amygdala | R | 44 | -28 | -6 | 3 | - | 5.31 |
| Middle temporal gyrus, temporal fusiform cortex, inferior temporal gyrus, planum polare | R | 44 | -15 | -16 | 3 | - | 5.23 |
| Frontal pole | R | 32 | 58 | 27 | 4 | 168 | 4.78 |
| Middle Frontal gyrus, precentral gyrus, inferior frontal gyrus | R | 40 | 12 | 30 | 5 | 452 | 4.47 |
| Lateral occipital cortex | R | 42 | -66 | 57 | 6 | 231 | 4.46 |
| Lateral occipital cortex | R | 51 | -62 | 51 | 6 | - | 3.9 |
| Frontal pole | L | -33 | 54 | 8 | 7 | 447 | 4.45 |
| Inferior temporal gyrus, middle temporal gyrus, | L | -50 | -15 | -28 | 8 | 489 | 4.37 |
| Inferior temporal gyrus | L | -48 | -20 | -38 | 8 | - | 3.83 |
| Middle frontal gyrus, precentral gyrus, superior frontal gyrus | R | 27 | 2 | 45 | 9 | 107 | 4.23 |
| Temporal fusiform cortex, parahippocampal gyrus | L | -27 | -33 | -22 | 10 | 160 | 4.23 |
| Frontal pole | R | 18 | 60 | -10 | 11 | 399 | 4.13 |
| Frontal pole | R | 28 | 64 | -3 | 11 | - | 4.04 |
| Frontal pole | R | 32 | 56 | 3 | 11 | - | 3.37 |
| Frontal pole, orbitofrontal cortex | R | 38 | 44 | -14 | 12 | 441 | 4.12 |
| Frontal pole, orbitofrontal cortex | R | 30 | 38 | -12 | 12 | - | 3.79 |
| Superior frontal gyrus, middle frontal gyrus | R | 26 | 24 | 51 | 13 | 188 | 4.08 |
| Supramarginal gyrus | R | 56 | -36 | 30 | 14 | 283 | 3.91 |
| Parietal operculum cortex, planum temporale | R | 50 | -24 | 15 | 14 | - | 3.87 |
| Parietal operculum cortex, supramarginal gyrus, planum temporale | R | 58 | -28 | 24 | 14 | - | 3.73 |

Note, Gray rows represent clusters significant at FDR *p* <.05, corrected, all other clusters are reported at whole-brain uncorrected, *p* <.001 with 100 voxels threshold. Bi = Bilateral; L = Left; R = Right.

Supplementary Table 2. Atrophy patterns in AD vs controls.

| Region | Side | MNI | | | Cluster | Voxels | *t* |
| --- | --- | --- | --- | --- | --- | --- | --- |
|  |  | X | Y | Z |  |  |  |
| Amygdala, hippocampus, pallidum, planum polare, putamen, temporal fusiform cortex | L | -16 | -10 | -14 | 1 | 9481 | 7.25 |
| Amygdala, hippocampus, orbitofrontal cortex, insula, parahippocampal gyrus, thalamus, | L | -28 | -10 | -15 | 1 | - | 6.6 |
| Amygdala, parahippocampal gyrus, hippocampus | L | -24 | 3 | -18 | 1 | - | 5.95 |
| Hippocampus, amygdala, parahippocampal gyrus, lingual gyrus, posterior cingulate gyrus | R | 26 | -9 | -14 | 2 | 11968 | 5.48 |
| Amygdala, parahippocampal gyrus, hippocampus, temporal pole, temporal fusiform cortex, putamen, insula | R | 16 | -2 | -18 | 2 | - | 5.41 |
| Temporal fusiform cortex, parahippocampal gyrus, | R | 38 | -28 | -18 | 2 | - | 5.37 |
| Lateral occipital cortex | L | -44 | -70 | 10 | 3 | 517 | 4.92 |
| Lateral occipital cortex, angular gyrus | L | -48 | -66 | 18 | 3 | - | 4.11 |
| Angular gyrus, middle temporal gyrus | L | -42 | -58 | 10 | 3 | - | 3.59 |
| Middle temporal gyrus, superior temporal gyrus | L | -66 | -40 | 2 | 4 | 706 | 4.86 |
| Thalamus | L | -3 | -15 | 6 | 5 | 1345 | 4.53 |
| Thalamus | R | 4 | -9 | 8 | 5 | - | 4.5 |
| Thalamus | L | -3 | 3 | -4 | 5 | - | 4.17 |
| Supramarginal gyrus, parietal operculum cortex, angular gyrus | R | 57 | -36 | 34 | 6 | 181 | 4.44 |
| Central opercular cortex, Heschl's gyrus, planum polare, planum temporale, | L | -54 | -12 | 8 | 7 | 159 | 4.29 |
| Inferior temporal gyrus, temporal occipital fusiform cortex, temporal fusiform cortex | R | 48 | -39 | -21 | 8 | 319 | 4.2 |
| Lateral occipital cortex, angular gyrus | R | 50 | -62 | 27 | 9 | 324 | 3.99 |
| Lateral occipital cortex, angular gyrus | R | 38 | -60 | 28 | 9 | - | 3.81 |
| Frontal operculum cortex, central opercular cortex, insula | L | -44 | 10 | 0 | 10 | 251 | 3.93 |
| Middle temporal gyrus, Superior temporal gyrus | L | 52 | -2 | -22 | 11 | 129 | 3.87 |
| Frontal pole | L | -42 | 46 | 4 | 12 | 133 | 3.78 |

Note, Gray rows represent clusters significant at FDR *p* <.05, corrected, all other clusters are reported at whole-brain uncorrected, p <.001 with 100 voxels threshold. Bi = Bilateral; L = Left; R = Right.

Supplementary Table 3. Scanner acquisition

|  | Scanner 1 | Scanner 2 | Scanner 3 |
| --- | --- | --- | --- |
| Site | Argentina | Argentina | Chile |
| Scanner | Philips Ingenia 3.0T with a standard head coil | 1.5 T Phillips Intera | SIEMENS Skyra 3.0T with a standard head coil |
| Repetition time (ms) | 8.3 | 7.49 | 2.4 |
| Echo time (ms) | 3.8 | 3.4 | 2 |
| Flip angle | 8° | 8° | 8° |
| Slices | 160 | 196 | 192 |
| Matrix dimension | 224x224x160 | 256x256x192 | 256x256x192 |
| Voxel size | 1mm x 1mm x 1mm | 1mm x 1mm x 1mm | 1mm x 1mm x 1mm |

Supplementary Table 4. Demographic information for participants included in neuroimaging analyses.

|  |  |  | Statistic | *p* |
| --- | --- | --- | --- | --- |
|  | bvFTD | Controls |  |  |
| Demographics | n = 24 | n = 21 |  |  |
| Age | 67.57 ± 12.12 | 66.24 ± 8.98 | -0.41 | .684 |
| Sex | 13:11 | 12:9 | 0.04 | .841 |
| Education | 13.77 ± 4.89 | 15.95 ± 2.46 | 1.86 | .072 |
|  | AD | Controls |  |  |
|  | n = 27 | n = 24 |  |  |
| Age | 74.74 ± 8.78 | 73.13 ± 4.53 | -0.81 | .422 |
| Sex (M:F) | 8:19 | 9:15 | 0.35 | .552 |
| Education | 12.37 ± 4.81 | 14.00 ± 3.15 | 1.41 | .165 |

**Supplementary results**

**
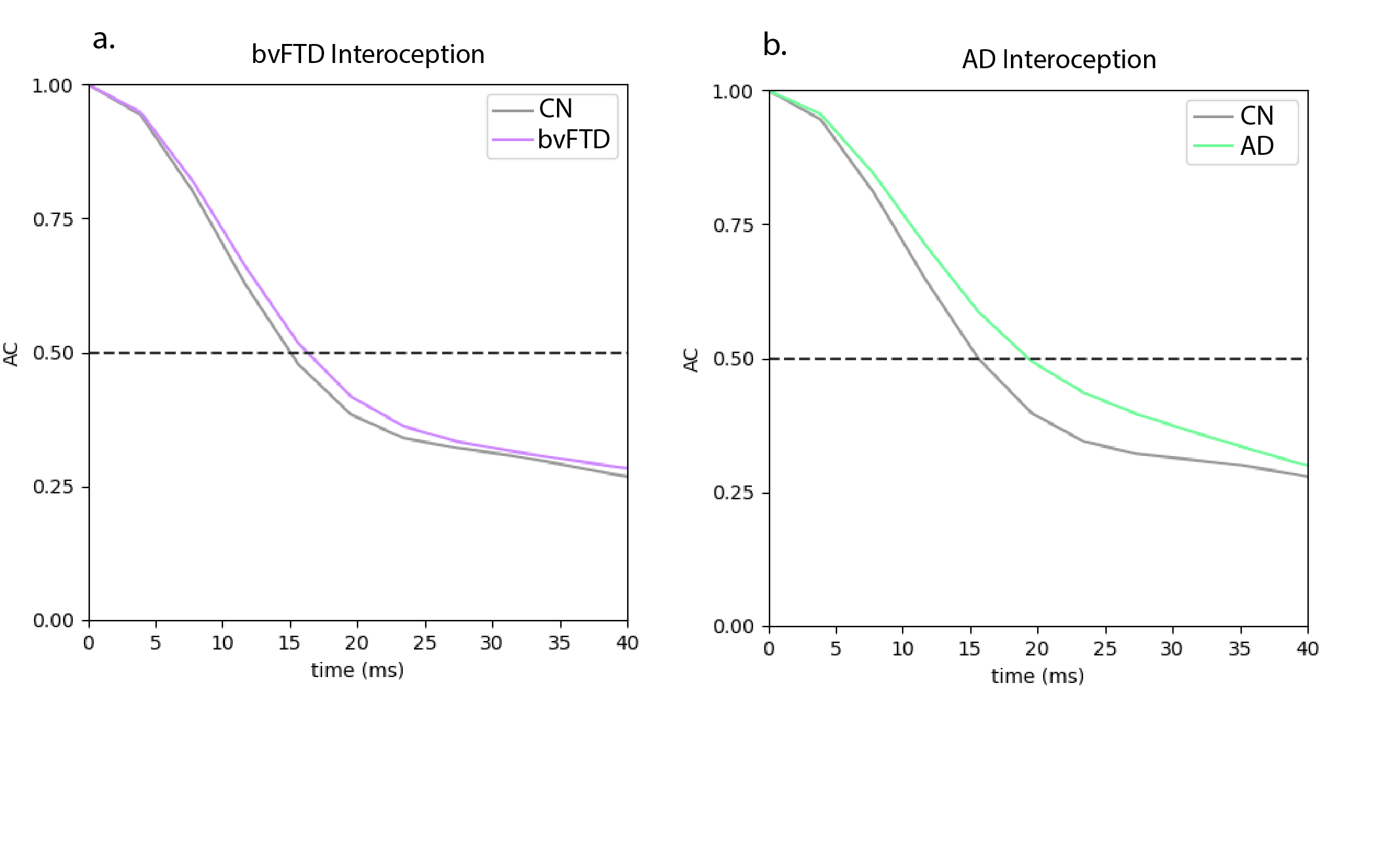
**

Supplementary Figure 1. Average autocorrelation function in interoception for a) bvFTD vs controls and b) for AD vs controls. AC = Autocorrelation.

Supplementary Table 5. Mini-SEA Raw versus imputed results.

|  | **Group** | | **Statistic** | ***p*** | **Hedges' g** |
| --- | --- | --- | --- | --- | --- |
|  | Controls | AD |  |  |  |
|  | N = 34 | N = 35 |  |  |  |
| Raw Mini-SEA Total (/30) | 24.90 ± 2.60 | 19.21± 3.68 | 5.41 | <.001 | 1.79 |
| Raw Mini-SEA Emotion (/15) | 12.04 ± 1.54 | 10.47 ± 2.19 | 2.92 | .003 | 0.81 |
| Raw Mini-SEA Faux pas (/15) | 12.93 ± 1.87 | 9.38 ± 2.44 | 4.98 | <.001 | 1.63 |
| Imp. Mini-SEA Total (/30) | 24.55 ± 2.29 | 20.30 ± 3.11 | 6.46 | <.001 | 1.54 |
| Imp. Mini-SEA Emotion (/15) | 11.78 ± 1.38 | 10.41 ± 1.93 | 3.33 | .001 | 0.79 |
| Imp. Mini-SEA Faux pas (/15) | 12.80 ± 1.56 | 9.88 ± 1.84 | 7.10 | <.001 | 1.69 |
|  | Controls | bvFTD |  |  |  |
|  | N = 32 | N = 31 |  |  |  |
| Raw Mini-SEA Total (/30) | 24.54 ± 2.20 | 20.47 ± 5.80 | 3.29 | .001 | 0.84 |
| Raw Mini-SEA Emotion (/15) | 11.79 ± 1.35 | 10.16 ± 2.64 | 2.71 | .01 | 0.72 |
| Raw Mini-SEA Faux pas (/15) | 13.01 ± 1.88 | 11.53 ± 2.25 | 2.29 | .01 | 0.69 |
| Imp. Mini-SEA Total (/30) | 24.50 ± 1.92 | 20.60 ± 5.56 | 3.69 | <.001 | 0.93 |
| Imp. Mini-SEA Emotion (/15) | 11.91 ± 1.58 | 10.23 ± 2.55 | 3.12 | .002 | 0.78 |
| Imp. Mini-SEA Faux pas (/15) | 12.78 ± 1.54 | 11.35 ± 2.24 | 2.94 | .002 | 0.74 |

Note. Missing data: MiniSEA Total, HC-AD tandem: HC = 12; AD = 18; HC-bvFTD tandem: HC = 14 ; bvFTD = 4; MiniSEA Emotion, HC-AD tandem: HC = 10; AD = 8; HC-bvFTD tandem: HC = 13; bvFTD = 4; MiniSEA Faux Pas, HC-AD tandem: HC = 12; AD = 18 ; HC-bvFTD tandem: HC = 13; bvFTD = 4.

Supplementary Table 6. Predictors of raw Mini-SEA Total and raw Mini-SEA Emotion data.

|  | Overall social cognition | | | Emotion recognition | | |
| --- | --- | --- | --- | --- | --- | --- |
| bvFTD-controls |  |  |  |  |  |  |
|  | b(SE) | *t* | *p* | b(SE) | *t* | *p* |
| Age | 0.08(0.08) | 1.02 | .315 | 0.03(0.03) | 1.01 | .318 |
| Sex | 0.93(1.40) | 0.66 | .512 | 0.46(0.59) | 0.79 | .437 |
| Education | 0.09(0.16) | 0.56 | .581 | -0.03(0.06) | -0.50 | .618 |
| Site | -2.31(1.49) | -1.55 | .130 | -1.45(0.63) | -2.30 | .027* |
| Diagnosis | -2.04(0.80) | -2.55 | .015* | -0.79(0.34) | -2.36 | .023* |
| ACW-50 | -1.38(0.53) | -2.62 | .013* | -0.69(0.22) | -3.09 | .004* |
| AD-controls |  |  |  |  |  |  |
| Age | 0.001(0.090) | 0.02 | .988 | -.002(0.045) | -0.05 | .958 |
| Sex | 0.24(1.06) | 0.23 | .821 | 1.00(0.61) | 1.65 | .105 |
| Education | 0.25(0.14) | 1.75 | .090 | 0.06(0.07) | 0.83 | .409 |
| Site | -0.99(1.32) | -0.75 | .459 | -0.29(0.69) | -0.42 | .677 |
| Diagnosis | -5.57(1.16) | -4.90 | <.001* | -1.69(0.62) | -2.73 | .009** |
| ACW-50 | 0.22(0.52) | 0.42 | .678 | 0.07(0.28) | 0.25 | .800 |

Supplementary Table 7. Predictors of cognitive performance, considering HEP.

|  | **Cognition** | | | | | **Executive functioning** | | | | |
| --- | --- | --- | --- | --- | --- | --- | --- | --- | --- | --- |
| **bvFTD v controls** | | | | | | | | | | |
| **Overall** | *F*(7, 52) = 9.29, *p*<.001, *R^2^* = 0.545 | | |  |  | *F*(7, 52) = 8.28, *p*<.001, *R^2^* = 0.527 | | |  |  |
|  | **b(SE)** | ***t*** | ***p*** | **b(SE)*** | **95%CI[L, U]*** | **b(SE)** | ***t*** | ***p*** | **b(SE)*** | **95%CI[L, U]*** |
| Constant | 24.57(2.26) | 10.86 | <.001 | 24.57(2.54) | [4.74, 14.98] | 23.82(4.68) | 5.09 | <.001 | 23.82(5.22) | [13.77, 34.39] |
| Age | 0.04(0.02) | 1.76 | 0.084 | 0.04(0.03) | [0.00, 0.11] | 0.07(0.05) | 1.44 | 0.157 | 0.07(0.06) | [-0.06, 0.19] |
| Sex | -0.44(0.50) | -0.87 | 0.380 | -0.44(0.53) | [-0.95, 1.13] | -1.64(1.04) | -1.58 | 0.119 | -1.64(1.01) | [-3.58, 0.34] |
| Education | 0.16(0.06) | 2.81 | 0.007 | 0.16(0.06) | [-0.14, 0.10] | 0.20(0.12) | 1.69 | 0.098 | 0.20 (0.12) | [-0.06, 0.43] |
| Site | -0.84(0.49) | -1.72 | 0.09 | -0.84(0.46) | [-2.20, -0.39] | -4.50(1.01) | -4.44 | <.001 | -4.50(1.10) | [-6.83, -2.45] |
| Diagnosis | -2.37(0.50) | -4.78 | <.001 | -2.37(0.51) | [-2.10, -0.08] | -4.57(1.02) | -4.46 | <.001 | -4.57(1.02) | [-6.64, -2.60] |
| ACW-50 | -0.55(0.19) | -2.86 | 0.006 | -0.55(0.23) | [-1.31, -0.42] | -0.67(0.40) | -1.68 | 0.099 | -0.67(0.46) | [-1.60, 0.22] |
| HEP | -1.71(2.03) | -0.84 | 0.402 | -1.71(1.65) | [-3.71, 2.73] | -2.11(4.19) | -0.50 | 0.617 | -2.11(3.65) | [-10.02, 4.60] |
| **AD v controls** | | | | | | | | | | |
| **Overall** | *F*(7,61) = 24.27, p <.001, *R^2^* = 0.736 | | |  |  | *F*(7,61) = 12.56, p <.001, *R^2^* = 0.590 | | |  |  |
|  | **b(SE)** | ***t*** | ***p*** | **b(SE)*** | **95%CI[L, U]*** | **b(SE)** | ***t*** | ***p*** | **b(SE)*** | **95%CI[L, U]*** |
| Constant | 30.97(2.78) | 11.14 | <0.001 | 30.94(2.87) | [25.06, 36.19] | 35.52(5.40) | 6.56 | <0.001 | 35.42(4.87) | [25.55, 45.93] |
| Age | -0.07(0.04) | -1.99 | 0.052 | -0.07(0.04) | [-0.15,0.009] | -0.17(0.07) | -2.44 | 0.018 | -0.17(0.07) | [-0.32, -0.05] |
| Sex | 0.72(0.48) | 1.49 | 0.141 | 0.72(0.52) | [-0.22, 1.81] | 0.31(0.94) | 0.34 | 0.738 | 0.31(0.96) | [-1.58, 2.16] |
| Education | 0.19(0.06) | 3.31 | 0.002 | 0.19(0.06) | [0.08, 0.32] | 0.15(0.11) | 1.35 | 0.182 | 0.15(0.12) | [-0.08, 0.39] |
| Site | -0.87(0.53) | -1.64 | 0.107 | -0.87(0.52) | [-1.92, 0.15] | -1.85(1.04) | -1.79 | 0.079 | -1.85(1.08) | [-3.84, 0.38] |
| Diagnosis | -4.98(0.48) | -10.31 | <0.001 | -4.98(0.45) | [-5.95, -4.17] | -6.97(0.94) | -7.43 | <0.001 | -6.97(0.98) | [-8.94, -5.09] |
| ACW-50 | -0.27(0.24) | -1.11 | 0.273 | -0.27(0.21) | [-0.71, 0.11] | -0.34(0.47) | -0.73 | 0.470 | -0.34(0.46) | [-1.27,0.53] |
| HEP | -0.06(1.67) | -0.04 | 0.972 | -0.06(1.51) | [-3.20, 2.91] | 4.45(3.25) | 1.37 | 0.176 | 4.48(3.43) | [-1.19, 12.04] |

Note. All *p* values are FDR-corrected. **p* < .05; ** *p*<.01. *** *p* <.001. b(SE)* represent bootstrapped coefficient and standard error values and 95%CI [L, U]* represent bootstrapped confidence intervals. All bootstrapping was performed using 5000 iterations. L = Lower CI, U = Upper CI.

Supplementary Table 8. Predictors of social cognition and emotion recognition performance, considering HEP.

|  | **Social cognition ^a^** | | | | | **Emotion recognition ^a^** | | | | |
| --- | --- | --- | --- | --- | --- | --- | --- | --- | --- | --- |
| **bvFTD v controls** | | | | | | | | | | |
| **Overall** | *F*(7, 52) = 4.75, *R^2^* = 0.39, p <.001 | | |  |  | *F*(7, 52) = 6.92, *R^2^* = 0.48, *p* <.001 | | |  |  |
|  | **b(SE)** | ***t*** | ***p*** | **b(SE)*** | **95%CI[L, U]*** | **b(SE)** | ***t*** | ***p*** | **b(SE)*** | **95%CI[L, U]*** |
| Constant | 17.72(4.98) | 3.56 | 0.001 | 17.72(4.72) | [8.69, 27.21] | 9.65(2.27) | 4.25 | <0.001 | 9.65(2.86) | [4.67, 15.12] |
| Age | 0.11(0.05) | 2.04 | 0.047 | 0.11(0.06) | [-0.02, 0.20] | 0.06(0.02) | 2.57 | 0.013 | 0.06(0.03) | [0.00, 0.11] |
| Sex | 0.52(1.11) | 0.468 | 0.641 | 0.52(1.16) | [-1.72, 2.88] | 0.08(0.50) | 0.16 | 0.873 | 0.08(0.52) | [-0.96, 1.11] |
| Education | 0.05(0.13) | 0.394 | 0.695 | 0.05(0.15) | [-0.26, 0.35] | -0.02(0.06) | -0.36 | 0.717 | -0.02(0.06) | [-0.14, 0.10] |
| Site | -1.38(1.08) | -1.28 | 0.207 | -1.38(0.97) | [-3.25,0.60] | -1.24(0.49) | -2.52 | 0.015 | -1.24(0.45) | [-2.18, -0.39] |
| Diagnosis | -3.03(1.09) | -2.78 | 0.008 | -3.03(0.94) | [-4.91, -1.25] | -1.09(0.50) | -2.20 | 0.032 | -1.09(0.51) | [-2.10, -0.08] |
| ACW-50 | -1.37(0.43) | -3.21 | 0.002 | -1.37(0.53) | [-2.50, -0.44] | -0.84(0.19) | -4.32 | <0.001 | -0.84(0.22) | [-1.29, -0.42] |
| HEP | -0.85(4.46) | -0.19 | 0.849 | -0.85(3.52) | [-8.97, 5.10] | -0.12(2.03) | -0.06 | 0.954 | -0.12(1.63) | [-3.67, 2.86] |
| **AD v controls** | | | | | | | | | | |
| **Overall** | *F*(7, 61) = 7.55, *R^2^* = 0.464, *p* <.001 | | |  |  | *F*(7, 61) = 7.55, *R^2^* = 0.229, *p* =0.020 | | |  |  |
|  | **b(SE)** | ***t*** | ***p*** | **b(SE)*** | **95%CI[L, U]*** | **b(SE)** | ***t*** | ***p*** | **b(SE)*** | **95%CI[L, U]*** |
| Constant | 22.13(3.99) | 5.54 | <0.001 | 22.13(4.63) | [10.14, 28.91] | 9.43(2.49) | 3.78 | <0.001 | 9.43(2.94) | [3.25, 14.61] |
| Age | -0.01(0.05) | -0.22 | 0.828 | -0.01(0.07) | [-0.11],0.15 | 0.01(0.03) | 0.26 | 0.794 | 0.01(0.04) | [-0.07, 0.09] |
| Sex | 0.69(0.69) | 0.99 | 0.326 | 0.69(0.70) | [-0.69,2.05] | 0.72(0.43) | 1.67 | 0.100 | 0.72(0.41) | [-0.07, 1.52] |
| Education | 0.20(0.09) | 2.35 | 0.022 | 0.20(0.06) | [0.07, 0.32] | 0.06(0.05) | 1.06 | 0.292 | 0.06(0.04) | [-0.03,0.14] |
| Site | -0.30(0.77) | -0.40 | 0.693 | -0.30(0.65) | [-1.62, 0.96] | -0.10(0.48) | -0.21 | 0.835 | -0.10(0.42) | [-0.89,0.73] |
| Diagnosis | -4.23(0.69) | -6.10 | <0.001 | -4.23(0.67) | [-5.58, -2.94] | -1.41(0.43) | -3.26 | 0.002 | -1.41(0.42) | [-2.26, -0.58] |
| ACW-50 | 0.16(0.35) | 0.47 | 0.643 | 0.16(0.31) | [-0.47, 0.76] | 0.04(0.22) | 0.20 | 0.845 | 0.04(0.19) | [-0.34, 0.42] |
| HEP | -0.02(2.40) | -0.006 | 0.995 | -0.02(2.29) | [-4.16, 5.10] | 1.44(1.50) | 0.96 | 0.341 | 1.44(1.33) | [-0.90, 4.43] |

Note. All *p* values are FDR-corrected. **p* < .05; ** *p*<.01. *** *p* <.001. b(SE)* represent bootstrapped coefficient and standard error values and 95%CI [L, U]* represent bootstrapped confidence intervals. All bootstrapping was performed using 5000 iterations. L = Lower CI, U = Upper CI. ^a^ Based on imputed values to handle missing data, raw regression models reported in Supplementary tables 5-6 for comparison.

**Interoceptive accuracy, heart-rate variability and HEP**

We investigated the relationship between the ACW-50 metric, interoceptive accuracy (as measured by the mean-distance index [(18)](https://paperpile.com/c/AnylZt/muQU). In brief, this metric compares the frequency of the participants' response to the frequency of their heartbeat, where a lower score represents a greater match between frequencies. This metric has been found to outperform other measures of interoceptive accuracy [(18)](https://paperpile.com/c/AnylZt/muQU), and has been investigated in neurodegeneration [(5,6,19)](https://paperpile.com/c/AnylZt/8dt2+2UGd+DU1j). We also investigated the relationship between the ACW-50 metric and HRV (measured by RR-interval and SD of the RR-interval)[(20)](https://paperpile.com/c/AnylZt/gyns).

**Group differences in interoceptive accuracy, heart rate variability, and HEP.**

bvFTD and AD patients had worse interoceptive accuracy than controls. No differences were observed in HRV RR-interval metrics between bvFTD or AD patients and controls. AD patients had greater HRV SD RR-ms than HC (*p* = .007), with a trend observed for greater HRV SD RR-ms in bvFTD patients than controls (*p* = 0.05), however, these values were within normal clinical range (50-100ms)(1). Both patient groups presented reduced HEP modulation in the interoceptive in comparison with exteroceptive condition (Supplementary Figure 2A), bvFTD patients showed reduced HEP modulation than controls in interoception only (see Supplementary Figure 2B). Whereas, reduced HEP modulation was observed in AD patients compared to controls in both interoception and exteroception (see Supplementary Figure 2B).

Supplementary Table 9. Heart-rate variability and interoceptive accuracy during interoception.

|  | **Group** | | **Statistic** | ***p*** | |
| --- | --- | --- | --- | --- | --- |
|  | **HC** | **AD** |  | |  |
|  | **N = 34** | **N = 35** |  | |  |
| HRV (R-R ms) | 949.8 ± 110.39 | 959.4 ± 144.01 | -0.295 | | .769 |
| HRV (SD R-R ms) | 48.1 ± 3.49 | 95.3 ± 6.06 | -2.637 | | .012 |
| Interoceptive accuracy (md) | 0.27 ± 0.22 | 0.55 ± 0.40 | -2.91 | | .007 |
|  | **HC** | **bvFTD** |  | |  |
|  | **N = 31** | **N = 31** |  | |  |
| HRV (R-R ms) | 910.0 ± 127.57 | 874.9 ± 136.3 | 1.00 | | 0.323 |
| HRV (SD R-R ms) | 44.0 ± 4.83 | 78.0 ± 7.43 | -2.02 | | 0.050 |
| Interoceptive accuracy (md) | 0.30 ± 0.18 | 0.64 ± 0.39 | -4.08 | | <0.001 |


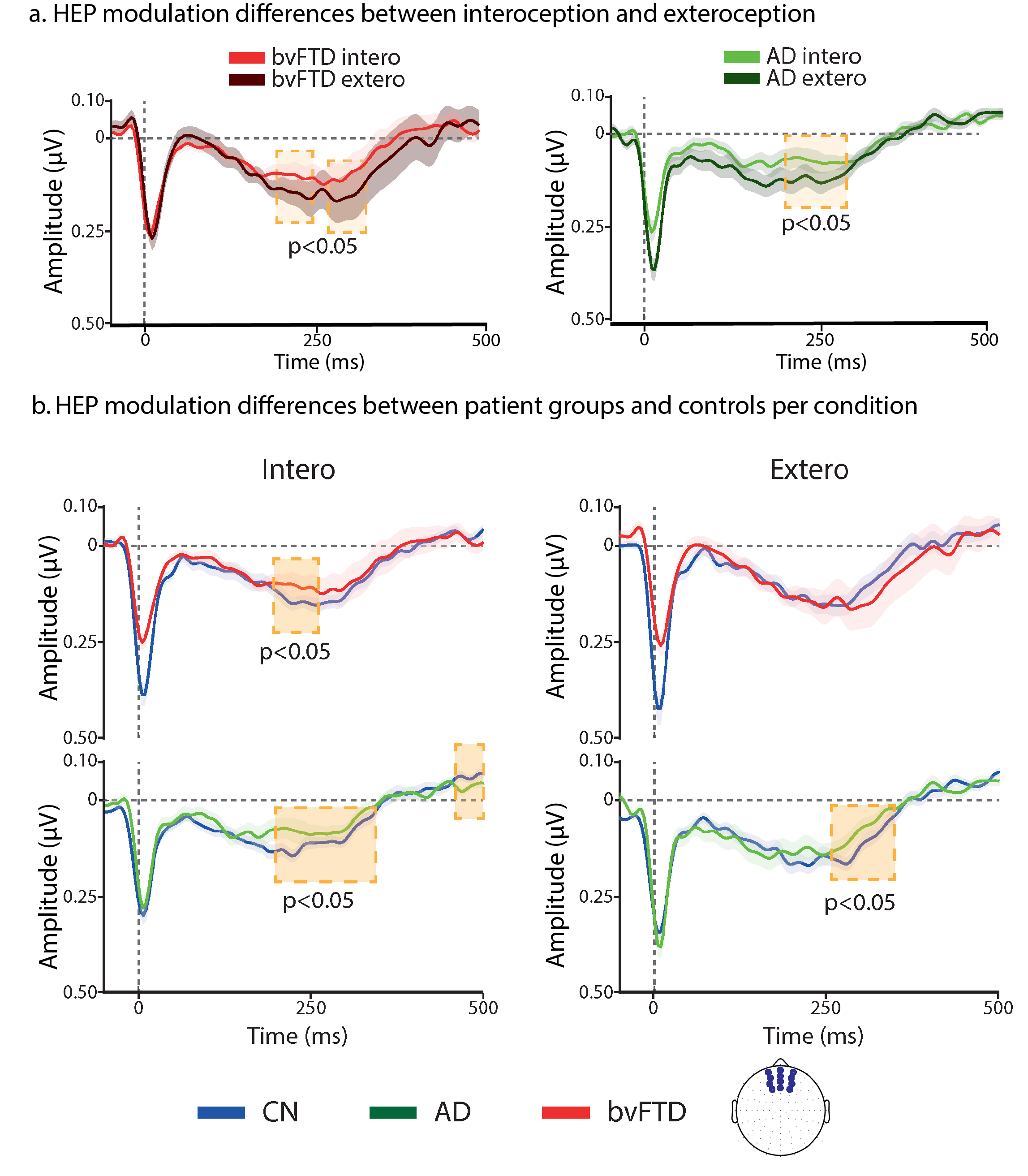


Supplementary Figure 2. Heart-evoked potential during interoception and exteroception a) within-groups and b) between groups. Point-by-point ERP comparison between patient groups and controls via Monte-Carlo permutation test. Orange boxes indicate p <.05 (a minimum extension of five clusters was selected as criteria to graph clusters). Shadowed bars represent standard error of the mean. Blue dots in channel location diagram illustrate electrodes included in the frontal ROI.

**Relationship between ACW-50, interoceptive accuracy, heart rate variability, and HEP.**

No association between ACW-50 and interoceptive accuracy was observed in bvFTD patients and controls combined, *r*(51) = .178, *p* = .11, or in AD patients and controls combined, *r*(49) = .164, *p* = .13. We also found no association between the ACW-50 metric and HRV in bvFTD patients and controls (HRV RR-interval: *r*(56) = -.150, *p* = .14; HRV SD-RR: *r*(56) = .126, *p* = .18) or in AD patients and controls (HRV RR-interval: *r*(62) = .103, *p* = .213; HRV SD-RR: *r*(62) = 0.025, *p* = .42). Finally, we found no association between ACW-50 and HEP modulation during interoception (average 200-500ms) in bvFTD patients and controls combined, *r*(58) = 0.042, *p* = 0.910, or in AD patients and controls combined, *r*(67) = 0.137, *p* = 0.259.

References

1. Nunan D, Sandercock GR, Brodie DA. A quantitative systematic review of normal values for short‐term heart rate variability in healthy adults. Pacing and clinical electrophysiology. 2010;33(11):1407-17.
